# Effect of Oven-Drying on the Recovery of Valuable Compounds from *Ulva rigida*, *Gracilaria* sp. and *Fucus vesiculosus*

**DOI:** 10.3390/md17020090

**Published:** 2019-02-01

**Authors:** Andreia F.R. Silva, Helena Abreu, Artur M.S. Silva, Susana M. Cardoso

**Affiliations:** 1 QOPNA & LAQV-REQUIMTE, Department of Chemistry, University of Aveiro, 3810-193 Aveiro, Portugal; afrs@ua.pt (A.F.R.S.); artur.silva@ua.pt (A.M.S.S.); 2 ALGAplus, Produção e Comercialização de Algas e seus Derivados, Lda., 3830-196 Ílhavo, Portugal; htabreu@algaplus.pt

**Keywords:** algae, drying, pigments, phenolic compounds, antioxidant activity, polysaccharides

## Abstract

The effect of oven-drying at 25, 40 and 60 °C was evaluated on three macroalgae of relevance in Europe, namely *Ulva rigida*, *Gracilaria* sp. and *Fucus vesiculosus*, with respect to quality aspects, including their potential to be exploited as a source of valuable compounds. Notably, as compared to freeze-drying, oven-drying at 25 °C promoted the extraction of chlorophylls and carotenoids from *U. rigida*, as well as those of phycoerythrin and chlorophyll *a* from *Gracilaria* sp., while 40 °C favored the recovery of fucoxanthin and pheophytin *a* from *F. vesiculosus*. On the other hand, the use of oven-drying had a negative impact on the extraction of phenolic compounds from this alga, also diminishing the antioxidant activity of the resulting extracts. Instead, the impact of oven-drying of raw material on the recovery of specific polysaccharides differed among the macroalgae. While the amounts of ulvans and fucoidans obtained from macroalgae dried at higher temperatures tended to be superior, the recovery of agar was not affected with the drying temperatures applied to *Gracilaria* sp. The overall results showed that oven-drying might serve as a good alternative to stabilize *Ulva rigida*, *Gracilaria* sp. and *Fucus vesiculosus*, especially if extraction of pigments and polysaccharides is aimed, thought the appropriate temperature applied must be adapted for each macroalgae.

## 1. Introduction

Seaweeds have been used in East Asia as food for centuries, whereas their exploitation in Western Countries was always associated with the extraction of polysaccharides such as carrageenan (mainly from *Kappaphycus* and *Eucheuma* origin) and agar (mainly from *Gracilaria* and *Gelidium* origin), which due to their stabilizing, water thickening, emulsifying and gelling properties have since ever a wide application in food industry. Due to attractive physicochemical and/or bioactive properties, their usage has also been increasing in non-food fields, including medicinal, pharmaceutical, cosmetic, paper and textile industries [1,2]. Particularly, in pharmaceuticals, carrageenan is used as an inactive excipient in emulsions, syrups and tablets, while their potential at the medicinal level is mainly related to the treatment of stomach ulcers, bowel problems (such as diarrhoea, dysentery), antiviral and anticoagulant [1,3,4]. In addition, highly sulphated agar has been suggested as a promising therapeutic agent in inflammatory bowel disease [5]. Moreover, in recent years, other algae-derived polysaccharides such as ulvans and fucoidans have been emerging as health-promoting agents [3,4]. The first refers to acidic water-soluble sulphated heteropolysaccharides mainly composed of uronic acids (glucuronic acid and iduronic acid), sulphated rhamnose, xylose and glucose, and are present in the cell walls of green seaweeds, particularly from the *Ulva* genus [6]. In turn, fucoidans are a complex series of polysaccharides mostly composed of sulphated fucose and of minor amounts of variable monosaccharides and are commonly found in the cell walls of brown seaweeds, where they are thought to have a protective role against the effects of desiccation [7]. Overall, these two sulphated polysaccharides have promising applications as bioactive compounds. E.g., the anti-thrombotic activity attributed iduronic acid from ulvans, render them value to be used in the synthesis of heparin fragments analogues [8]. In the same way, low molecular weight fucoidan has been used to mimic the biologic activity of heparan sulphates [9]. Moreover, rhamnan, rhamnose and oligomers from desulphated Monostroma ulvans has been patented for the treatment of gastric ulcers [10]. In addition to that, these polysaccharides have been reported to exert anti-tumoral, immunomodulatory, anti-hyperlipidemic, anti-coagulant and antioxidant activities [5,6], which are expected to boost their applications in future.

Pigments such as chlorophylls, β-carotene, lutein, fucoxanthin and phycobiliproteins are also valuable compounds [1,7] and frequently applied in the industry. Their range of applications are naturally associated with their colors, although bioactive properties also assume high relevance, especially for carotenoids. Indeed, lutein is presently marked as an ingredient in oral tablets and in many multivitamin supplements, due to its claimed role in eye diseases prevention, cancer, diabetes and heart diseases [11]. In the same line, β-carotene supplements are largely used as the so-called oral sun protectants, since it promotes the production of vitamin A [12]. Moreover, fucoxanthin i.e., a characteristic pigment of brown seaweeds, is known to exert remarkable biological properties, including antiobesity, antidiabetic, anti-inflammatory, anticancer properties and protection from hepatic, cardiovascular and cerebrovascular damaging events, which give it huge application as nutraceutical and pharmaceuticals [13]. Besides being critical in photosynthesis, chlorophylls and phycobiliproteins can be applied as natural colorants e.g., in foods and simultaneously act as antioxidant additives, and able to act as bioactive compounds [1,12]. In particular, phycobiliproteins have been reported to be endowed of antioxidant, anticancerous, neuroprotective, anti-inflammatory, hepatoprotective and hypocholesterolemic abilities [14].

Likewise, phenolic compounds, which are general accepted as antioxidant agents, represent high-attractive seaweed components for application in health-promoting products. Among those, phlorotannins i.e., polymers formed through C-C and/or C-O-C oxidative coupling of phloroglucinol, are phenolic compounds particularly abundant in brown macroalgae [12,13]. In addition to antioxidant effects (scavenging of reactive oxygen species and/or enhancement of intracellular antioxidant defenses), these compounds have demonstrated to exert antidiabetic properties through acarbose-like activity, stimulation of adipocytes glucose uptake and protection of pancreatic cells against high-glucose oxidative stress, as well as anti-inflammatory effects through inhibition of several pro-inflammatory mediators, among other beneficial activities that render them great potential for application in numerous therapeutic approaches [15].

Seaweeds are naturally highly perishable because of their high water content (60 to 94%) [1], hence being mostly marketable as dried products. In the next years, the increasing seaweeds market demands will cause an exponential production of biomass and the need of fast and controlled drying methods. Dried seaweeds traded in the food, cosmetics and feed markets are mainly dried in two manners: sun/open-air drying and forced air tunnels. Oven-drying and freeze-drying processes are normally used in experimental studies to assess biomass composition or, in the case of the latter, in very high-value applications [16]. Naturally, regardless sun/open-air drying is more sustainable and low cost, it is conditioned by climacteric conditions and day length, which constitute a hygiene risk, also compromising the quality of the products in terms of colour and nutritional composition. Contrary, drying in forced air tunnels has in general a high processing capacity and is able of generating dehydrated products with great extended shelf-life, being already widely applied in industry [17,18]. Still, regardless its convenience, this technique faces various constraints, ranging from the economical to the quality levels of products. It is known that water removal from the algae causes a deformation that might lead to the degradation of its matrix and consequently affect the functionalities and integrity of their cell walls and membranes, as well as of its components. Naturally, the extent of these effects depends on distinct factors, including the seaweed itself and the applied temperature [19].

To our knowledge, the effect of oven-drying on valuable compounds of *Ulva*, *Gracilaria* and *Fucus* genre has been scarcely studied. Robic et al. [20] evaluated the impact of oven-drying at 50 and 70 °C, on the yield of recovery and physicochemical features of ulvans, obtained from wild *Ulva rotundata* of France origin, while Rodrigues et al. [21] focused the rehydration ratio and water holding ability of *Ulva lactuca* (unspecific origin) oven-dried at 30 and 40 °C. Lastly, recently, Uribe et al. [22] studied the impact of different drying methods, including oven-drying at 70 °C, on the surface color and phytochemical content and amino acid and fatty acid profiles of wild *Ulva* spp. from Chile. In turn, Tello-Ireland and collaborators [23] have previously analysed the effect of moderate-to-high oven-drying temperatures (40, 50, 60 and 70 °C) on distinct physicochemical parameters of *Gracilaria chilensis* from Chile, as compared to fresh algae, although no information has been delivered regarding the use of low temperatures. Moreover, the effect of oven-drying in *Fucus* is limited to the study of Moreira et al. [24], whom tested the use of temperatures in the range of 35–75 °C on a wild *F. vesiculosus* sample from Galicia and its effects on the color of the resulting powders and on the yield of antioxidants and alginates in aqueous extracts, obtained by ultrasound-assisted extraction methodology.

In this context, the present study is intended to gather complementary data with respect to the impact of oven-drying (temperature at 25, 40 and 60 °C versus freeze-drying) in distinct parameters of three macroalgae of relevance in Europe, namely *Ulva rigida* (green), *Gracilaria* sp. (red) and *Fucus vesiculosus* (brown), with emphasis on their specific valuable compounds. Note that, in opposition to the previous works, which used wild macroalgae, the herein used macroalgae were obtained in a land-based cultivation system, more specifically under an integrated multi-trophic aquaculture regime (IMTA), which is considered as a sustainable cultivation system with controlled conditions and adequate for market supply.

## 2. Results and Discussion

### 2.1. Moisture Content

**Figure 1:**
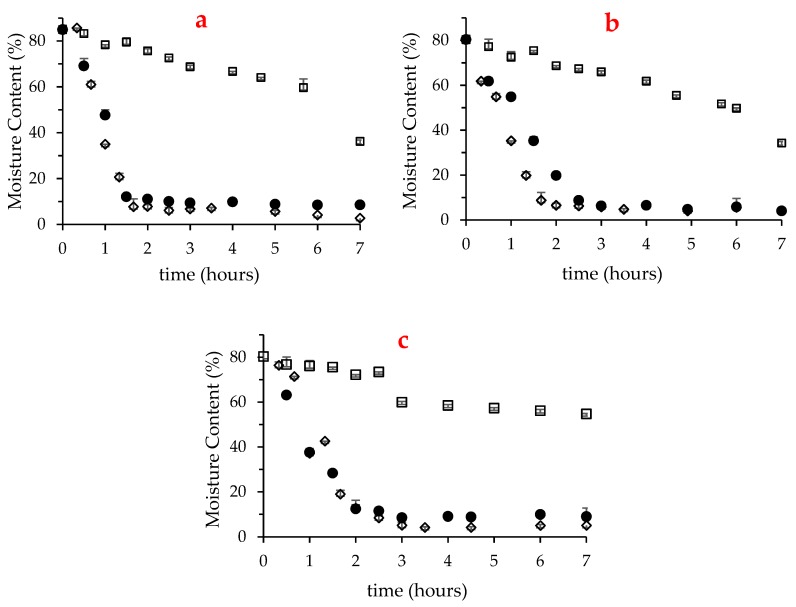
Variation of the moisture content of *U. rigida* (**a**), *Gracilaria* sp. *(***b**) and *F. vesiculosus* (**c**) over seven hours in oven-drying at 25 °C (□), 40 °C (●) and 60 °C (◊).

Fresh macroalgae presented high moisture content (84.7% in *U. rigida* and about 80% in *Gracilaria* sp. and *F. vesiculosus*), which as expected, decreased along the drying treatment. Figure 1 depicts the variation of moisture content for the first 7 h of drying in *U. rigida*, *Gracilaria* sp. and *F. vesiculosus*, at 25, 40 and 60 °C. Notably, this period was not sufficient to dry any of the three macroalgae at 25 °C, although compared with *F. vesiculosus*, the moisture content of the other two species was substanciatlly reduced, reaching 34–35%. Consistent with this, at 25 °C, *U. rigida* and *Gracilaria* sp. reached drynesss (moisture of 10%) after about 15 h, while *F. vesiculosus* required approximatly 24 h (results not shown). Despite dryness at 60 °C was fast, the longer drying period of *F. vesiculosus* was also noted at this temperature (2 h in *F. vesiculosus* vs. 1.6 h in the two other seaweeds).

The comparison of the herein gathered results with literature is not a simple task, since the drying parameters, such as the scale of the equipment and its load, ventilation velocity and temperature applied, as well as macroalgae specificities (e.g., thallus thickness, length and additional structures), cause unavoidable changes in the drying rate of macroalgaes. Indeed, widely dispersed drying conditions and results were reported earlier. E.g., Uribe et al. [22] observed that the convective drying at 2.0 m/s of four hundred grams of *Ulva* sp. at 70 °C took 2 h to dry. Instead, Gupta et al. [25] followed the drying of five grams of brown macroalgae *Himanthalia elongata* (at 2.0 ± 0.1 m/s), which were reported to achieve dryness after 5 and 8 h at 40 and 25 °C, respectively. In turn, in the work of Moreira et al. [24], aproximately three kilograms of *F. vesiculosus* required 25 h to dry at 35 °C and at least 20 h at 70 and 80 °C.

### 2.2. Surface Color

Color is a critical quality attribute and is commonly affected during processing and storage of raw natural products [26]. In particular, changes in surface color caused by thermal processes may occur due to distinct enzymatic and non-enzymatic reactions. E.g., the chemical reaction of polyphenol oxidase produces oxidized forms of phenolics compounds that act as intermediates of brown pigments. In addition, non-enzymatic Maillard reactions occurring between reducing sugars and amino acids also contribute to brown pigmentation [26]. Moreover, chlorophylls easily degrade to gray-brown compounds such as pheophytin or pheophorbide through chemical and enzymatic reactions [27] and carotenoids (e.g., lutein and β-carotene) undergo isomerization and oxidation reactions also impacting their colors [28,29].

The effect of oven-drying on the surface color of *U. rigida*, *Gracilaria* sp. and *F. vesiculosus* was evaluated, as compared to fresh samples, using the color coordinates defined by the International Commission on Illumination (CIE L*a*b*) (Table 1). Note that in this system, a* takes negative or positive values for greenish or reddish colors, respectively, whereas b* takes negative values for the bluish tonalities, and positive values for yellowish ones. L* is an approximate measurement of luminosity, which is the property according to which each color can be considered as equivalent to a member of the greyscale, between black and white [30]. In addition, based on CIE L*a*b* coordinates, the browning index (BI, defined as brown color purity) of the samples can be estimated.

Oven-drying of *U. rigida* caused considerable changes in color coordinates, particularly for 60 °C. In general, compared to fresh macroalgae, those subjected to oven-drying tended to have lower luminosity upon rehydration, indicating that they were darker. This fact might also partially influence the greenish tonalities of the oven-dried samples, as compared to the control ones. Nonetheless, one must highlight that b* was the most affected coordinate in *U. rigida*, showing ∆b* (i.e., variation between the treated sample and control) of 1.25, 3.55 and 6.96 (at 25, 40 and 60 °C, respectively), with respect to control. The intensification of the yellow tonality caused by oven-drying has been previously reported to occur in the green microalgae *Arthrospira Spirulina* sp. and in *Spirogyra* sp. and theorized by the authors to be due to chlorophylls degradation [31,32].

In opposition to *U. rigida*, oven-drying of *Gracilaria* sp. caused a higher impact in a* than in b* coordinate, with ∆a* values ranging from 4.2 to 6. The increase of redness tonality in oven-dried (40–70 °C) macroalgae with respect to fresh samples has been previously recorded by Tello-Ireland et al. [23] for *Gracilaria chilenses*, whom attributed differences to changes on the algae pigments, including phycobiliproteins, red phycoerythrin, carotenes, lutein, and zeaxanthin. Consistent with our results, these authors also reported an increment of the macroalgae luminosity as a function of thermal treatment [23].

Regarding *F. vesiculosus*, our results indicated that the oven-drying at 25 °C caused no significant changes in the a* parameter in relation to control samples. Moreover, this coordinate was not affected at high temperatures as well, while yellow tonalities and luminosity increased (∆b* values of 1.43 and 4.68 and ∆L* of 2.15 and 4.41, for temperatures of 40 and 60 °C, respectively). These observations agree with the study of Moreira and collaborators [24], whom verified an increase of b* and L* coordinates in milled *F. vesiculosus* after oven-drying at 60 and 75 °C, relatively to 50 and 35 °C, possibly as a consequence of browning reactions. Curiously, our results also allowed to conclude that regardless the observable variations in the browning index of *F. vesiculosus* at 40 °C, this was less than half of those of *Gracilaria* sp. and *U. rigida*, suggesting that at that temperature, the green and red macroalgae are more susceptible to non-enzymatic browning reactions (∆BI of 4.39 in *F. vesiculosus* vs. 10-11 in the two other algae). The lower variation of BI in *F. vesiculosus* was even observed at 60 °C, particularly with respect to *Gracilaria* sp. (∆BI of 21 and 25, respectively), a fact that might be associated with its inferior protein content.

Total color difference (ΔE*) is a key parameter for the assessment of magnitude of color differences between control and treated samples. Differences in perceivable color can be analytically classified as very distinct (ΔE* > 3), small differences (1.5 < ΔE* < 3) and undetectable differences (1.5 < ΔE*) by non-experimented observers [33]. Considering this parameter, in general, the herein collected data indicated that *Gracilaria* sp. and *F. vesiculosus* are clearly distinct regarding the impact of oven-drying on the color surface changes. Indeed, while significant differences (ΔE* > 3.0) in *F. vesiculosus* were only visible for 60 °C, a ΔE* superior to 6 was registered for all oven-dried *Gracilaria* sp. On the other hand, *U. rigida* showed an intermediate behavior, with small differences detected even if the samples were dried at low temperature (25 °C), which turned into clear differences for dried algae at 40 and 60 °C. Still, please note that overall, the color of *U. rigida* was the most affected when the temperature of 60 °C was applied (ΔE* of 9.17).

### 2.3. Water Retention Capacity (WRC)

**Figure 2:**
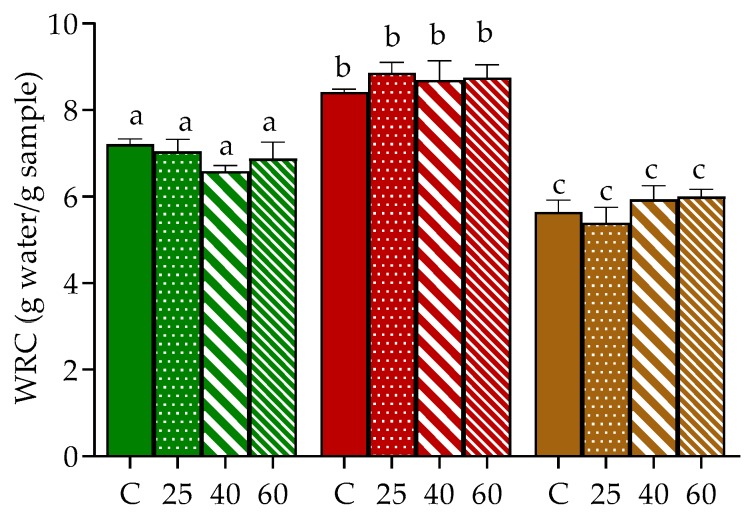
Water retention capacity of *U. rigida* (green), *Gracilaria* sp. (red) and *F. vesiculosus* (brown), dried by freeze-drying (C) or oven-drying at 25 °C (25), 40 °C (40) and 60 °C (60). Values are presented as mean ± standard deviation. For each seaweed, same letters indicate no significant differences (*p* > 0.05) according to Tukey´s test.

Hydration properties of seaweed’s tissues, namely WRC, is generally related to the content of polysaccharides, especially dietary fiber, and to protein that might link to cell wall polysaccharides [34]. Moreover, their rehydration ability, as for dried products in general, can offer some clues about the degree of tissue damage caused by drying and pre-treatments [35]. In our study, the WRC of oven-dried *U. rigida*, *Gracilaria* sp. and *F. vesiculosus* was evaluated and compared to samples stabilized by freeze-drying (Figure 2), since this latter technique is considered one of the mildest drying methodologies, able to preserve most of the original qualities of the biological materials [16].

Naturally, the WRC of the three samples varied, being higher in *Gracilaria* sp., followed by *U. rigida* and *F. vesiculosus* (8.4, 7.2 and 5.6 g water/g algae, respectively, in control). Similar results in WRC were recently reported for *Gracilaria* sp. by our group, albeit in that study, higher values were found in the case of *U. rigida* and *F. vesiculosus* [36]. Discrepancies between the present results and those previously reported for the same algae species are probably associated with differences in the chemical composition (e.g., fiber and protein contents) of the macroalgae batch. Consistent with this, Benjama and Masniyom [34] reported WRC values for *Ulva pertusa* and *Ulva intestinalis* of 8.08 and 13.9 g/g dw (mean values), respectively, with higher values corresponding to the macroalgae sample with higher protein and fiber contents.

In general, no significant differences were registered between the WRC of freeze-dried and oven-dried samples, which may suggest the imperceptible impact of hot drying conditions on the structure of the cell wall of *U. rigida*, *Gracilaria* sp. and *F. vesiculosus*. To our knowledge, the effect of oven-drying in the WRC of *Ulva* and *Fucus* genus was not previously described, hence impairing data comparison. However, the herein gathered results for *Gracilaria* sp. seem to differ from those reported for *Gracilaria chilenses* since in their study, authors have registered a significant decrement of WRC between samples dried at 40 and 60 °C [23]. Once again, differences might be due to the distinct composition of the cell wall in the macroalgae samples, among other factors.

### 2.4. Extractability of Valuable Compounds

#### 2.4.1. Pigments

Variations in the surface color of the macroalgae may reflect, in some cases, possible changes in their pigments. Still, the recovery of pigments from the macroalgae matrix does not only dependent on their integrality, but also on factors such as the type of solvent, time of extraction and sample conditions (if fresh, dried, freeze or milled) [37,38]. Hence, this work also intended to evaluate the impact of oven-drying on the recovery of relevant pigments from the three macroalgae, namely chlorophylls and carotenoids, which are known to be present in the three algae Filo, as well as on phycobiliproteins, which are exclusive from red macroalgae. The conditions of extraction (e.g., type of solvent and mass:volume ratio) were selected based on literature data [38,39] and preliminary assays of our group (unpublished data).

As expected, the acetone extracts obtained from *U. rigida* were mainly rich in chlorophylls *a* and *b* (Chl *b* exclusive of this Phylum [40]), also containing relevant quantities of the carotenoid lutein (Table 2). On the other hand, the chlorophyll profile of *F. vesiculosus* was dominated by chlorophyll *a* and its derivate pheophytin *a*, in addition to the carotenoids lutein, β-carotene and fucoxanthin, being the latter the most prominent of this class. Instead, chlorophyll *a* was the unique pigment detected through chromatographic analysis of the acetone extract of *Gracilaria* sp. origin. Moreover, in agreement with the color of this macroalgae, phycoerythrin was the major soluble phycobiliprotein detected in the aqueous extracts.

Clearly, the impact of oven-drying on the recovery of pigments differed among the three macroalgae. Overall, it is curious that with exception of Chl *a* in *F. vesiculosus*, this was not maximal in freeze-dried. In fact, in *U. rigida*, the greatest amounts of chlorophylls and lutein were recovered in samples dried at 25 °C, while levels of chlorophylls obtained from samples dried at 40 and 60 °C were close to those of the control. Moreover, drying at these two temperatures also allowed to obtain a superior amount of lutein in comparison to the freeze-drying process. Drying at 25 °C was also the most favorable condition for the recovery of pigments in *Gracilaria* sp., in particular for Chl *a* and phycoerythrin (PE). Similarly, to *U. rigida*, the raising of the drying temperature tended to reduce the recovery of pigments (chlorophyll and phycobiliproteins), although levels were not significantly lowered with respect to the control. To our knowledge, the recovery of pigments from *Gracilaria* genus has only been focused by Tello-Ireland et al. [23], who compared the extraction of PE and phycocyanin (PC) in oven-dried samples (40 to 70 °C) with respect to fresh samples. Although it is not possible to direct compare our results with those previously described by the authors, the combining of both studies suggests that thermal drying of *Gracilaria* at mild temperatures (up to 40 °C) do not impair the recovery of phycobiliproteins, in comparison to freeze-dried or fresh samples. Still, one must highlight that in opposition to our work, those authors registered a clear decrement in the recovery of phycobiliproteins when raising the drying temperature from 40 to 60 °C, while this trend in our study was observed at inferior temperatures (between 25 and 40 °C). Differences can of course be due to distinct factors, including the *Gracilaria* species under study. Nevertheless, it is clear that the application of high temperatures can compromise the yield of extraction of phycobiliproteins, perhaps due to their protein nature and consequence denaturation, or even due to morphologic structural changes of seaweed triggered by strong heat and dehydration, as it occurs in other seafood [41,42].

On the other hand, the pattern of pigments recovery in *F. vesiculosus* was clearly distinct from those of green and red macroalgae. Please note that the acetone extracts of this alga were dominated by fucoxanthin, which is known to be the main responsible pigment for the color of brown macroalgae [43]. Moreover, one should also highlight the presence of high quantities pheophytin *a* (characteristic tonality olive brown), which can also contribute to its color. The recovery of these two pigments from *F. vesiculosus* dried at 25 °C or freeze-dried were similar, regardless the levels of less representative carotenoids (lutein and β-carotene) and Chl *a* were superior in the latter, possibly reflecting their enhanced accessibility to solvents. Levels of these compounds obtained from samples dried at 40 °C were, however, close to those of the control condition, and most important, this temperature allowed for the obtaining of maximal amounts of the main chlorophyll derivative (phaeophytin *a*) and of fucoxanthin. Once again, compared to 40 °C, drying at 60 °C negatively impacted the recovery of these pigments. Previous studies focused on the extraction of fucoxanthin from other algae species, as a function of drying temperatures. In this context, Stévant et al. [44] reported that the drying temperature of *Saccharina latissima* at 70 °C potentized the extraction of fucoxanthin (compared to drying at 25 °C and freeze-drying processes), although the authors admit that this trend was not significant due to the variability between treatment groups. In addition, *Sargassum* sp. dried at 90 °C also resulted in extracts with higher fucoxanthin content, compared to those obtained from seaweeds dried at 65 and 40 °C, a fact that was theorized by the authors to be caused by the increased break down of cell walls, which allow the release of more fucoxanthin from the seaweed matrix [45]. Hence, it is feasible to think that oven-drying might promote the extraction of fucoxanthin from brown macroalgae, in comparison to freeze-drying. The most appropriate temperature for fucoxanthin extraction must, however, be adapted to the species in focus, since variable cell-wall compositions are found among them.

Overall, our results also seem to indicate that while thermal drying at moderate temperatures (25 °C for *U. rigida* and *Gracilaria* sp., and up to 40 °C in *F. vesiculosus*) do not significantly degrade pigments and might even improve the accessibility of solvents to the macroalgae cellular compartments, as compared to non-destructive drying processes, namely freeze-drying. The use of higher temperatures of drying, namely 40 °C in *U. rigida* and *Gracilaria* sp., or 60 °C in all the three macroalgae, result in the degradation of such compounds. Please note that the superior resistance of *F. vesiculosus* to pigments degradation herein reported can partially be associated with the results of surface color, since the latter also indicated perceptible changes in *U. rigida* and *Gracilaria* sp. at lower temperatures (25 and 40 °C), while those in *F. vesiculosus* were visually undetected at those temperature conditions (Table 1).

#### 2.4.2. Phenolic Compounds and Antioxidant Activity

The amounts of total phenolic compounds (TPC) recovered from *U. rigida* and *Gracilaria* sp. were much lower than that extracted from *F. vesiculosus* (approximately 1 vs. 11 mg EGA/g seaweed, in samples stabilized by freeze-drying) (Table 3), which agrees with the predominance of these compounds in brown macroalgae with respect to Chlorophyta and Rhodophyta [1]. Notably, in opposition to chlorophylls and carotenoids, the extraction of these compounds was extensively diminished by oven-drying, even at 25 °C. These results are also consistent with those of other authors that pointed freeze-drying as a suitable stabilization technique, able to afford the best extraction yields of phlorotannins in Fucaceae (such as *Lessonia spicata* and *Sacharina latissima*) among several techniques, including freezing, silica-drying and oven-drying [44,46].

As expected, the reduced amount of phenolics in the extracts obtained from oven-dried samples was accompanied by a decrease in their antioxidant capacity, with values of IC_50_ varying from 0.06 to 0.09 mg/mL, in control and oven-dried samples, respectively. These results agree in part with those of Moreira et al. [24], whom found a linear correlation between TPC and antioxidant activity (using DPPH· radical scavenging activity test) in aqueous extracts of *F. vesiculosus*. It is, however, relevant to note that in contrast to our study, those authors showed an inversed correlation between the increasing of drying temperature (35 to 75 °C) and the TPC of the extracts. Differences can naturally occur due to the distinct factors, including the divergent oven-drying and the extraction conditions.

The effect of oven-drying in *U. rigida* and *Gracilaria* sp. phenolics was not as drastic as in *F. vesiculosus*, since their recovery was close in samples dried at 25 °C, compared to the control. Moreover, regardless levels of phenolics were tendentially reduced for incremented drying temperatures, in particular for 60 °C, there were no significant differences. This tendency in *U. rigida* did not impact the antioxidant capacity of the extracts, however, those of *Gracilaria* sp. were reduced for 40 and 60 °C. These results agree with those described by Tello et al. [23], whom described a decrement of TPC and antioxidant activity in aqueous extracts from *Gracilaria chilensis*, when varying the drying temperature from 40 to 50 °C [23]. To our knowledge, there is no previous studies focusing the effect of distinct oven-drying temperatures on the recovery of phenolic compounds from *Ulva*. Yet, Uribe and collaborators [22] also reported that TPC recovered from *Ulva* sp. was higher in freeze-drying samples, in comparison to those stabilized at 70 °C.

### 2.5. Polysaccharides

**Figure 3:**
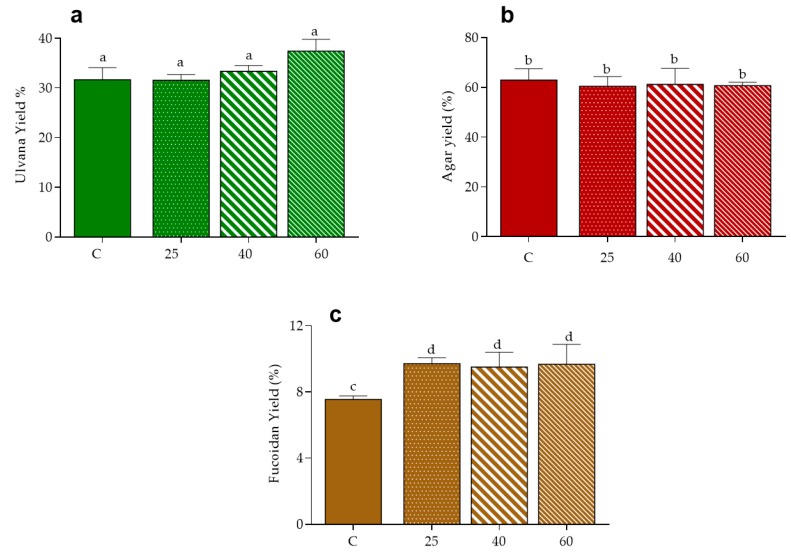
Yield of polysaccharides from seaweeds: ulvan from *U. rigida* (**a**), agar from *Gracilaria* sp. (**b**) and fucoidan from *F. vesiculosus* (**c**). Data are presented as mean ± standard deviation. Values are presented as mean ± standard deviation. Same letters indicate no significant differences (*p* > 0.05) according to Tukey’s test.

The effect of drying on the extraction of polysaccharides was evaluated in the three macroalgae, according with their composition specificities i.e., ulvans in *U. rigida*, agar in *Gracilaria* sp. and fucoidans in *F. vesiculosus* [47]. As shown in Figure 3, the yield of recovery of the ulvan fraction in control conditions was close to 32% and this was similar to that obtained from samples dried at 25 °C, suggesting that oven-drying at low temperatures do not affect the extraction of this polysaccharides in *U. rigida*. Values tended, however, to increase for higher drying, reaching a mean value of 37% at 60 °C. Note that the increased recovery of ulvans caused by high temperature of drying of the raw material has been previously reported for *U. rotundata* stabilized at 50 and 70 °C, a fact that authors hypothesized to the associated with a more efficient inhibition of endogenous enzymatic breakdown of these polysaccharides [48].

In turn, the yield of agar extract from *Gracilaria* sp. was about 60% in all tested conditions, i.e., did not vary with the application of oven-drying in comparison to freeze-drying, neither with variation of the air temperature. Curiously, these results differ from those of Tello-Ireland et al. [23], whom reported an increment of agar extraction yield when raising the temperature of drying of *G. chilensis* from 40 to 70 °C. It is, however, evident that values of recovery in such study (28 to 40 g/100 g dried plant at 70 °C) are very different from the ones herein reported. Hence one must recall that besides extraction conditions, other factors like macroalgae species and seasonality of seaweed harvest may influence agar extracted yield [40,49].

Regarding the fucoidan extract, the yield of recovery from *F. vesiculosus* was improved in oven-dried samples, in comparison to those of control (9.5–9.7 vs. 7.5%, respectively), suggesting that the application of thermal drying increases the extractability of these polysaccharides, independently of its magnitude, in the range of 25 to 60 °C.

Note that the levels of the extracted polysaccharides (*Gracilaria* sp. > *U. rigida* > *F. vesiculosus*) might be associated with their respective WRC (Figure 2).

## 3. Materials and Methods

### 3.1. Chemicals

Acetone, ethanol, methanol, sodium carbonate, calcium chloride and gallic acid were purchased from Panreac (Barcelona, Spain). Butylated hydroxytoluene (BHT) were purchased from Acros (Hampton, NH, USA). Folin reagent, 2,2′-azino-bis(3-ethylbenzothiazoline-6-sulphonic acid) (ABTS), ascorbic acid, chlorophyll *a*, chlorophyll *b*, lutein, β-carotene and fucoxanthin were purchased from Sigma-Aldrich (St. Louis, MO, USA). Solvents including ethanol, methanol and acetonitrile of high-performance liquid chromatography (HPLC) purity were purchased from Lab-Scan (Lisbon, Portugal).

### 3.2. Sample Collection and Drying Processing

The seaweeds *Gracilaria* sp., *F. vesiculosus* and *U. rigida* were cultivated in a land-based integrated multi-trophic aquaculture (IMTA) system at ALGAplus Lda, a company based in Aveiro district, Portugal, specialized in seaweed cultivation and their commercialization into the food, cosmetics and feed markets. After collection, the macroalgae were washed with sterilized seawater followed by centrifugation to remove excess water. Drying studies (Section 2.1) were performed with 300 g of fresh seaweeds, which were spread in aluminium trays with holes and dried in laboratory oven (BD, Binder, NY, USA), at 25, 40 and 60 °C, with air velocity of approximately 3.52 ± 0.27 m/s, as measured by an anemoscope (UT361, UNI-T, Dongguan, China). The residual moisture of the dried samples was estimated through drying at 105 °C overnight to constant weight. The oven-dried samples selected for the remaining studies (Section 2.2, Section 2.3 and Section 2.4) corresponded to those for which the moisture value reached close to 10%. Freeze-dried samples, as obtained in a VirTis^®^ BenchTop™ “K” Series freeze-dryer (VirTis, Warminster, PA, USA), contained a residual moisture of 9.4, 7.4 and 8.1% (for *U. rigida*, *Gracilaria* sp. and *F. vesiculosus*, respectively).

### 3.3. Surface Color and Browning Index

To estimate the effect of oven-drying on surface color, the whole seaweeds were hydrated in distillate water for 15 min and the excess water was removed with absorbent paper. Surface color was determined with a colorimeter (CM 2300d, Konica Minolta, Japan) through coordinates CIELAB a* (+ red, − green), b* (+ yellow, − blue) and L* (lightness) [33] and the color difference was calculated by the equation:

(1) ΔE*=[(a*−a0*)2+(b*−b0*)2+(L*−L0*)2]12

where a*, b* and L* correspond to the coordinates of processed macroalgae (dried), while ${a_{0}}^{*}$, ${b_{0}}^{*}$ and ${L_{0}}^{*}$ correspond to those of non-processed samples i.e., fresh seaweeds. These coordinates were used in browning index (BI) determination [50], through the:

(2) $$\mathrm{BI}=\frac{\left[100\times \left(X-0.31\right)\right]}{0.17}\;\mathrm{where}\;X=\frac{\left(a^{*}+1.75\times L^{*}\right)}{\left(5.645\times L^{*}+a^{*}-3.012\times b^{*}\right)}$$

### 3.4. Water Retention Capacity (WRC)

Approximately 10 mL of deslilated water was added to 200 mg of powder sample and kept in contact to hydratation for 30 min under magnetic stirred. Then, the samples were centrifuged at 6000 rpm for 30 min and the supernatant was discarded by decantation. WRC was estimated according equation:

(3) $$\frac{\left(m_{\mathrm{hydrated}}-m_{\mathrm{dried}}\right)}{m_{\mathrm{dried}}}$$

where m_hydrated_ is the mass of sample hydrated after centrifugation, and m_dried_ is the mass of sample dried. WRC was express in grams of water by grams of sample.

### 3.5. Extration and Quantification of Pigments

#### 3.5.1. Chlorophylls, Carotenoides and Fucoxantin

Clorophylls and carotenoids were extracted with acetone with 0.1% of butylated hidroxytoluene (BHT), for 24 h, using powdered samples and a ratio mass:volume ratio of 1:100 and 1:20, in the case of *U. rigida* and *Gracilaria* sp./*F. vesiculosus*, respectively. The extraction solution was filtered throught a nylon filter of 0.22 µm (Whatman™, Buckinghamshire, UK) and further analysed by UHPLC-DAD-ESI-MS^n^. This analysis was performed on an Ultimate 3000 (Dionex Co., San Jose, CA, USA) apparatus, composed of a quaternary pump, an autosampler, an ultimate 3000 Diode Array Detector (Dionex Co., San Jose, CA, USA) and an automatic thermostatic column compartment. The column used was a 100 mm length, 2.1 mm i.d., 1.9 μm particle diameter, end-capped Hypersil Gold C18 column (Thermo Scientific, San Jose, CA, USA) and its temperature was maintained at 30 °C. Gradient elution was carried out with 0.1% of formic acid (*v*/*v*) (solvent A) and acetonitrile:methanol (70/30) (solvent B). The solvent gradient consisted of a series of linear gradients, starting with 15–28% of solvent B over 3.9 min, increasing to 100% in 2.2 min and maintaining this value up to 25 min, followed by the return to the initial conditions, with a total running time of 20 min. The flow rate used was 200 mL × min^−1^ and the UV–Vis spectral data for all peaks were accumulated in the range 200–700 nm. The chromatographic profiles were recorded at 655 and 450 nm.

The mass spectrometer used was a Thermo LTQ XL (Thermo Scientific, San Jose, CA, USA) ion trap MS equipped with an ESI source. Control and data acquisition were carried out with the Thermo Xcalibur Qual Browser data system (Thermo Scientific). Nitrogen above 99% purity was used and the gas pressure was 520 kPa (75 psi). MS analysis was performance with negative voltage of 5 kV and an electrospray ionization (ESI) capillary temperature of 275 °C with 20–25 arbitrary units of energy collisions in fragmentations.

For quantitative analysis, the limits of detection and quantification were calculated from the parameters of the calibration curves obtained by an injection of known concentrations of the exact (chlorophyll *a*, chlorophyll *b*, lutein, β-carotene and fucoxanthin) or structurally-related standard (chlorophyll *a* in the case of pheophytin *a* quantification) compounds.

#### 3.5.2. Phycobiliproteins

The phycobiliproteins were extracted from *Gracilaria* sp. according the methodology of Martins et al. [51], with some adaptations. The seaweed was frozen in liquid nitrogen, milled and the resulted powder was extracted with sodium phosphate buffer 0.1 M, pH = 6.8, in proportion of 0.1 (m:v) for 30 min under magnetic stirring, at room temperature and protected from light. The resultant extract was centrifuged at 6000 rpm at 4 °C for 30 min. The absorbance profile of the supernatant, between 400 and 700 rpm, was used to calculate the concentration of phycobiliproteins allophycocyanin (APC), phycocyanin (PC) and phycoerythrin (PE) by the equations of Kursar et al. (1983), expressed in mg/g macroalgae:

$$\begin{array}{c} \mathrm{APC}=181.3\times A_{651}-22.3\times A_{614}\\ \mathrm{PC}=151.1\times A_{614}-99.1\times A_{651} \end{array}$$

(4) $$\mathrm{PE}=155.8\times A_{498.5}-40.0\times A_{614}-10.5\times A_{651}$$

### 3.6. Obtaining of Methanolic Extracts

20 mL of methanol was added to 1g of seaweed freeze-dried powder and the mixture was stirred for 24 h at room temperature, under light protection, followed by centrifugation at 6000 rpm for 10 min and filtration of the supernatant through a G4 sintered plate filter. The filtrate was storage at −18 °C until analysis.

#### 3.6.1. Total Phenolics Compounds (TPC)

Total phenolic content was determined using Folin-Ciocalteu by the general methodology previously reported [52], with minor adaptations. Briefly, in a 96-well plate 15 μL of Folin Ciocalteu reagent was added to 65 μL of deionized water followed by the addition of 10 μL of sample/standard and 5 min of incubation at room temperature. After that, 150 μL of 7% (*w*/*v*) sodium carbonate (Na_2_CO_3_) solution was added to each well and the plate was incubated for 60 min at 30 °C. Gallic acid was used as standard and TPC concentration was expressed in mg of Gallic acid equivalents (GAE) of g of seaweed.

#### 3.6.2. Antioxidant Activity

The antioxidant activity of extracts was measured in terms of radical-scavenging ability of radical ABTS·^+^, as previously described [53]. Several concentrations of extracts/standard (50 μL) were added to 250 µL of diluted ABTS·^+^ solution. After 20 min of incubation in dark conditions, the absorption at 734 nm was measured using an ELx800 microplate reader (BioTek, Winooski, VT, USA). The percentage of inhibition was calculated using the equation:

(5) $$\mathrm{ABTSscavenging}\;\mathrm{activity}\left(\%\right)=\left({\mathrm{Abs}}_{\mathrm{control}}-{\mathrm{Abs}}_{\mathrm{sample}}\right)/\;{\mathrm{Abs}}_{\mathrm{control}}\times 100$$

where Abs_control_ is the absorbance of ABTS radical the control without extract addition and Abs_sample_ is the absorbance of ABTS radical with extract. The results were expressed as IC_50_ (concentration of the extract able to inhibit the 50% of the ABTS·^+^) of each extract. Ascorbic acid was used as a positive control for comparison.

### 3.7. Polysaccharides Extraction

#### 3.7.1. Ulvans

The extraction of ulvans from *U. rigida* was performed in accordance to the procedure of Pankiewicz et al. [54], with some modifications. 2 g of seaweed powder were extracted dichloromethane-acetone (1:1, *v*/*v*) for 2 h in a Soxhlet system for pigments removal. The resulting dried residue (0.5 g) was extracted with 15 mL of distillate water at 80 °C, for 7 h under stirring, followed by centrifugation at 6000 rpm for 10 min and filtration through a G4 sintered plate filter. The filtrate was treated with α-amylase (3 µL, 3000 U/mL, Megazyme, Bray, Ireland) during 60 min at 20 °C, in order to remove starch. After enzymatic digestion, ethanol was added (1:4, *v*/*v*) to precipitate ulvans and the precipitate was recovered by filtration and dried with ethanol 96% (*v*/*v*) and acetone.

#### 3.7.2. Agar

Agar extraction was performed in *Gracilaria* sp., following the method of Kumar and Fotedar [28]. The seaweed (135 g) was hydrated in distilled water (20 mL) at room temperature for 1.5 h, followed by extraction at 90 °C for 3 h. Following, the mixture was filtered through cotton gaze and the liquid fraction was cooled, frozen to promote agar precipitation, thawed and dried in plates at 60 °C until dryness.

#### 3.7.3. Fucoidan

The fucoidan-rich fraction was obtained from 0.5 g of *F. vesiculosus*, according to the general methodology described by Wang and Chen [55]. The seaweed powder was pre-treated with 10 mL of ethanol 85% for approximately 5 h, followed by centrifugation and washing with acetone. The dried residue was then extracted with distillate water at 65 ± 5 °C for 1 h, and the mixture was centrifuged at 6000 rpm for 10 min. Subsequently, CaCl_2_ solution was added up to 2% (*v*/*v*) and kept overnight at 4 °C, to promote alginates precipitation. The alginate fraction was removed by centrifugation, while ethanol absolute was added to the supernatant to reach 70% (*v*/*v*), and let at 4 °C for 12 h, in order to promote polysaccharides precipitation, which were then recovered by centrifugation, followed by drying at 60 °C.

### 3.8. Statistical Analysis

All experiments were performed with at least three independent assays. Data were statistically analyzed by a trial version of GraphPad Prism 6.01 software (OriginLab Corporation, Northampton, MA, USA) using one-way ANOVA analysis and Tukey’s multiple comparisons test.

## 4. Conclusions

The impact of different oven-drying temperatures (25, 40 and 60 °C) applied to *U. rigida*, *Gracilaria* sp. and *F. vesiculosus* were investigated for several aspects, namely surface color, water holding capacity, the recovery of pigments, polyphenols and polysaccharides. Among the three macroalgae, *F. vesiculosus* was shown to be the most resistant to color changes, with perceptible differences only appearing at 60 °C, while *Gracilaria* sp. was the most sensible one.

Notably, oven-drying at 25 °C showed to be more favorable than freeze-drying with regard to the extraction of chlorophylls and carotenoids from *U. rigida*, as well as phycoerythrin and chlorophyll *a* from *Gracilaria* sp. In turn, the maximal recovery of the main pigments of *F. vesiculosus* was achieved in samples dried at higher temperature, namely at 40 °C, a fact that can partially be associated with its improved resistance to color changes. Moreover, oven-drying allowed to obtain equal amounts of agar from *Gracilaria* sp., and tended to increase the yield of recovery of ulvans and fucoidans from *U. rigida* and *F. vesiculosus*, respectively. In turn, the best conditions of drying with respect to the extraction of phenolic compounds and antioxidant ability were obtained for freeze-drying samples, especially in *F. vesiculosos* or, at lower temperatures of drying. This data suggests that distinct drying conditions should be applied to macroalgae, which should take into consideration their final commercial exploitation.

## Figures and Tables

**Table 1:** Surface color coordinates of fresh and oven-dried *U. rigida, Gracilaria* sp. and *F. vesiculosus*.

Drying Condition	CIELAB	*U. rigida*	*Gracilaria* sp.	*F. vesiculosus*
Fresh	a*	−10.18 ± 0.52	4.86 ± 0.47 ^d^	1.12 ± 0.33 ^f^
	b*	43.65 ± 0.98 ^a^	3.77 ± 0.50 ^d^	2.56 ± 1.08 ^f^
	L*	72.18 ± 1.13 ^a^	14.32 ± 0.91 ^d^	21.00 ± 1.24 ^f^
	BI	65.17	70.94	18.65
	ΔE*	-	-	-
25 °C	a*	−11.86 ± 0.37 ^a^	9.73 ± 0.80 ^e^	0.65 ± 0.25 ^f^
	b*	44.89 ± 0.90 ^a^	5.16 ± 0.81 ^e^	2.27 ± 0.65 ^f^
	L*	72.07 ± 1.16^ab^	18.52 ± 0.84 ^e^	21.46 ± 0.79 ^f^
	BI	65.11	96.20	14.25
	ΔE*	2.09	6.59	0.72
40 °C	a*	−11.60 ± 0.25 ^a^	9.09 ± 1.71 ^e^	0.91 ± 0.40 ^f^
	b*	47.20 ± 1.74 ^b^	4.03 ± 0.61 ^de^	3.99 ± 0.94 ^g^
	L*	70.20 ± 1.19 ^b^	18.65 ± 1.46 ^e^	23.15 ± 1.05 ^g^
	BI	75.16	81.21	23.04
	ΔE*	4.30	6.05	2.59
60 °C	a*	−13.57 ± 0.34 ^b^	10.09 ± 0.71 ^f^	1.46 ± 0.50 ^f^
	b*	50.61 ± 0.64 ^c^	4.72 ± 0.46 ^de^	7.24 ± 0.99 ^h^
	L*	67.28 ± 1.99 ^c^	18.49 ± 1.88 ^e^	25.41 ± 1.12 ^h^
	BI	87.15	94.60	39.76
	ΔE*	9.17	6.76	6.43

Values are presented as mean ± standard deviation. Color difference (ΔE*) of a specific dried sample was calculated with respect to the fresh macroalgae. Different letters (a,b,c,d,e,f,h,g) in the same column (for a specific colorimetric coordinate) indicate significant differences (*p* < 0.05) according to Tukey’s test.

**Table 2:** Main extracted pigments (µg/mg of dry seaweed) from *U. rigida, Gracilaria* sp. and *F. vesiculosus* dried in different conditions.

Seaweed	Drying Condition	Chlorophylls		Carotenoids		
		Chl *a*	Chl *b*	Lut		
*U. rigida*	C	2.66 ± 0.11 ^a^	0.88 ± 0.08 ^a^	0.42 ± 0.06 ^a^		
	25	4.55 ± 0.31 ^b^	1.21 ± 0.10 ^b^	1.20 ± 0.09 ^b^		
	40	2.58 ± 0.19 ^c^	0.68 ± 0.01 ^a^	0.66 ± 0.02 ^a^		
	60	2.67 ± 0.14 ^c^	0.83 ± 0.03 ^ab^	0.78 ± 0.06 ^a^		
		Chl *a*	Phe *a*	Lut	βCart	Fucx
*F. vesiculosus*	C	1.06 ± 0.07 ^d^	2.41 ± 0.11 ^d^	0.18 ± 0.02 ^d^	0.47 ± 0.04 ^d^	0.85 ± 0.06 ^d^
	25	0.24 ± 0.02 ^e^	2.50 ± 0.28 ^d^	0.03 ± 0.00 ^d^	0.24 ± 0.01 ^d^	0.78 ± 0.06 ^d^
	40	0.71 ± 0.03 ^f^	2.96 ± 0.25 ^e^	0.15 ± 0.02 ^d^	0.55 ± 0.05 ^d^	1.79 ± 0.08 ^c^
	60	0.35 ± 0.25 ^ef^	2.99 ± 0.03 ^e^	0.07 ± 0.00 ^d^	0.35 ± 0.02 ^d^	0.91 ± 0.05 ^d^
		Chlorophylls		Phycobiliprotein		
		Chl *a*		PE	PC	APC
*Gracilaria* sp.	C	0.34 ± 0.11 ^gh^		1.37 ± 0.09 ^g^	0.45 ± 0.02 ^g^	0.34 ± 0.02 ^g^
	25	0.82 ± 0.01 ^h^		2.37 ± 0.19 ^h^	0.69 ± 0.09 ^g^	0.45 ± 0.06 ^g^
	40	0.14 ± 0.06 ^g^		1.47 ± 0.13 ^g^	0.42 ± 0.06 ^h^	0.46 ± 0.08 ^g^
	60	0.30 ± 0.02 ^g^		1.19 ± 0.13 ^g^	0.51 ± 0.19 ^h^	0.32 ± 0.08 ^g^

C (freeze-dried), 25 (dried at 25 °C), 40 (dried at 40 °C), 60 (dried at 60 °C); Chlorophyll *a* (Chl *a*), Chlorophyll *b* (Chl *b*), Pheophytin *a* (Phe *a*), Lutein (Lut), β-carotene (βcart), Fucoxanthin (Fucx), Phycocyanin (PC), Phycoerythrin (PE), Allophycocyanin (APC). Values are presented as mean ± standard deviation. Different letters (a,b,c,d,e,f,g,h) in the same column (for a specific colorimetric coordinate) indicate significant differences (*p* < 0.05) according to Tukey’s test.

**Table 3:** Content in total phenolic compounds (TPC) and antioxidant activity (IC_50_) of methanol extracts of *U. rigida*, *Gracilaria* sp. and *F. vesiculosus* dried in different conditions.

Drying Condition	*U. rigida*		*Gracilaria* sp.		*F. vesiculosus*	
	TPC	IC_50_	TPC	IC_50_	TPC	IC_50_
**C**	0.93 ± 0.13 ^a^	0.47 ± 0.15 ^a^	1.39 ± 0.48 ^b^	0.80 ± 0.09 ^b^	11.0 ± 0.28 ^d^	0.06 ± 0.01 ^d^
**25**	1.24 ± 0.31 ^a^	0.34 ± 0.17 ^a^	1.46 ± 0.42 ^b^	0.79 ± 0.13 ^b^	6.72 ± 1.41 ^e^	0.09 ± 0.02 ^d^
**40**	0.96 ± 0.32 ^a^	0.43 ± 0.08 ^a^	1.24 ± 0.43 ^b^	1.70 ± 0.53 ^c^	7.28 ± 1.35 ^e^	0.09 ± 0.01 ^d^
**60**	0.74 ± 0.13 ^a^	0.31 ± 0.06 ^a^	1.11 ± 0.34 ^b^	1.89 ± 0.72 ^c^	7.20 ± 0.45 ^e^	0.09 ± 0.06 ^d^

C (freeze-dried), 25 (dried at 25 °C), 40 (dried at 40 °C), 60 (dried at 60 °C); TPC expressed in mg equivalents of gallic acid (EGA)/g of seaweed; IC_50_ represents the extract concentration that is required for scavenging 50% of ABTS radical (in mg of dry matter/mL of extract); Values are presented as mean ± standard deviation. Different letters (a,b,c,d,e) in the same column indicate significant differences (*p* < 0.05) according to Tukey’s test.
